# An Engineered Factor Va Prevents Bleeding Induced by Anticoagulant wt Activated Protein C

**DOI:** 10.1371/journal.pone.0104304

**Published:** 2014-08-15

**Authors:** Annette von Drygalski, Vikas Bhat, Andrew J. Gale, Laurent Burnier, Thomas J. Cramer, John H. Griffin, Laurent O. Mosnier

**Affiliations:** 1 The Scripps Research Institute, Dept of Molecular and Experimental Medicine, La Jolla, California, United States of America; 2 University of California San Diego, Dept of Medicine, Div of Hematology/Oncology, San Diego, California, United States of America; Emory University School of Medicine, United States of America

## Abstract

**Objective:**

An increased risk of bleeding is observed in patients receiving activated protein C (APC), which may be a limiting factor for the application of novel APC therapies. Since APC's therapeutic effects often require its cytoprotective activities on cells but not APC's anticoagulant activities, an agent that specifically antagonizes APC's anticoagulant effects but not its cytoprotective effects could provide an effective means to control concerns for risk of bleeding. We hypothesized that ^super^FVa, an engineered activated FVa-variant that restores hemostasis in hemophilia could reduce APC-induced bleeding.

**Approach and Results:**

^Super^FVa was engineered with mutations of the APC cleavage sites (Arg506/306/679Gln) and a disulfide bond (Cys609-Cys1691) between the A2 and A3 domains, which augment its biological activity and cause high resistance to APC. ^Super^FVa normalized APC-prolonged clotting times and restored APC-suppressed thrombin generation in human and murine plasma at concentrations where wild-type (wt) FVa did not show effects. Following intravenous injection of APC into BALB/c mice, addition to whole blood ex vivo of ^super^FVa but not wt-FVa significantly normalized whole blood clotting. Blood loss following tail clip or liver laceration was significantly reduced when ^super^FVa was administered intravenously to BALB/c mice prior to intravenous APC-treatment. Furthermore, ^super^FVa abolished mortality (∼50%) associated with excessive bleeding following liver laceration in mice treated with APC.

**Conclusions:**

Our results provide proof of concept that ^super^FVa is effective in preventing APC-induced bleeding and may provide therapeutic benefits as a prohemostatic agent in various situations where bleeding is a serious risk.

## Introduction

Increased risk of bleeding is observed in patients receiving therapy with a variety of anticoagulants and there is a general unmet need for prohemostatic agents that reduce bleeding risks or that can be used as an antidote when bleeding occurs. We hypothesize that ^super^FVa, an engineered FVa-variant that efficiently normalizes hemostasis in hemophilia A [1], fits the criteria for a prohemostatic biologic and may provide beneficial effects for bleeding associated with treatment of activated protein C (APC). As an anticoagulant enzyme, APC proteolytically inactivates activated factor V (FVa) and FVIIIa. APC rapidly inactivates FVa via proteolytic cleavage at Arg506 followed by a slower cleavage at Arg306. Since FVa enhances prothrombinase ∼10,000-fold inactivation of FVa by APC effectively shuts down thrombin formation [2]–[4].

In some clinical trials, treatment of severe sepsis with wt- APC therapy (Xigris, Eli Lilly, Indianapolis, IN, USA), was associated with an increased risk of serious bleeding in ∼3.5% of patients [5], [6]. Therefore, APC could not be administrated when conditions such a disseminated intravascular coagulation, thrombocytopenia or liver failure with coagulopathy coexisted. However, in most animal models of inflammatory injury and septic disease where APC was beneficial, APC's cytoprotective effects were responsible for the protective effects of APC therapy, whereas its anticoagulant effects were neither required nor contributing [7], [8]. Thus, wt-APC's anticoagulant activities and associated risk of bleeding may be a limiting factor for potential novel indications and next generation APC therapies. The availability of an APC-anticoagulant specific antidote or reversal agent that does not affect APC's cytoprotective activities seems therefore highly desirable.

^Super^FVa was engineered to improve hemostasis in hemophilia and reduce bleeding by increasing the efficiency of FVa to augment thrombin generation. Mutations of the APC cleavage sites (Arg506/306/679Gln) increase its biological activity, whereas an engineered disulfide bond (Cys609-Cys1691) between the A2 and A3 domains enhance its pharmacological efficiency due to a ∼3-fold enhanced specific activity compared to wt-FVa. Because of these modifications, ^super^FVa was found to be highly resistant to APC with superior hemostatic properties in hemophilia *in vitro* and *in vivo* compared to wt-FVa and other FVa variants [1]. We hypothesized that ^super^FVa, as an engineered FVa-variant that potently normalizes hemostasis in hemophilia, fits the criteria for a prohemostatic biologic that can reduce bleeding induced by wt-APC.

## Materials and Methods

### Recombinant factor (F)V mutants

Recombinant wt-FV and ^super^FV were made on a B-domain deleted S2183A platform and purified from conditioned media of stable transfected BHK cells by a combination of affinity chromatography using anti-FV 3B1 and HV5101 monoclonal antibodies as described [1], [9], [10]. FV protein concentration was determined by absorbance at 280 nm using FV ε_1%_ = 15.4 [9] and ELISA (Enzyme Research Laboratories, South Bend, Louisiana, USA) according to manufacturer's instructions. FV proteins were activated with 2 nM thrombin for 20 minutes at 37°C in prothrombinase buffer (50 mM HEPES, 150 mM NaCl, 0.5% BSA, 5 mM CaCl_2_ and 0.1 mM MnCl_2_). Activation was terminated by the addition of 1.1 molar equivalent of hirudin (Calbiochem). Protein purity, disulfide linkage and detailed protein characterization done as described [1].

### Prothrombinase assays

Prothrombinase assays were performed as described [9]. Briefly, FVa and phospholipid vesicles were mixed and 15 µL aliquots were added to 10 µL FXa, followed by 10 µL of prothrombin in prothrombinase buffer (final concentrations: 1.42 nM FXa, 28 pM FVa, 22 µM phospholipid vesicles, and 0.42 µM prothrombin). After 2.5 minutes, the reaction was quenched by addition to 50 µL Hepes Buffered Saline (HBS) containing 10 mM EDTA, 0.5% BSA, pH 8.2. After addition of 35 µL Pefachrome TH (0.6 mM) thrombin formation was assessed by measuring the change in absorbance at 405 nm using a VersaMax Microplate reader (Molecular Devices).

### Dosing of FVa variants

As with FVIII, FVa variant dosing for in vivo experiments was based on units/kg. For FVa dosing units were determined as prothrombinase cofactor activity, whereby the activity of 20 nM wild-type FVa (approximate FV plasma concentration) was defined as one Unit [1]. Dosing by activity is the usual method for clinical administration of clotting factors, which takes into account variations of specific activity in biological material. The specific activity of the ^super^FVa used here was 2.8-fold higher compared to wt-FVa, which translates into a 2.8-fold lower protein concentration for ^super^FVa when injected into mice. For these experiments 5 and 25 units/mouse corresponded to 0.7 and 3.5 mg/kg ^super^FVa.

### Activated Protein C (APC)

Recombinant human (rh) APC (Xigris, Eli Lilly and Co, Indianapolis, Indiana, USA), plasma-derived APC, and murine recombinant APC (rmAPC) were used as indicated and prepared as described previously [11]–[14].

### Thrombin generation assays

Endogenous thrombin potential (ETP) assays were performed as described [8]. Briefly, FVa, rhFVIIa (NovoSeven; Novo Nordisk, Bagsvaerd, Denmark), 4-Factor Prothrombin Complex Concentrate (Prothromplex Total S-TIM 4,Baxter; Vienna, Austria) or saline were added to 50% (v/v) human (George King Bio-Medical, Overland Park, Kansas, USA) or murine plasma (BALB/c; Bioreclamation, Westbury, New York, USA) supplemented with 1.45 µM corn trypsin inhibitor (Haematologic Technologies, Essex Junction, Vermont, USA), 10 mM CaCl_2_, 10 µM phospholipid vesicles (80% phosphatidylcholine, 20% phosphatidylserine), 0.2 pM soluble tissue factor (Innovin, Dade Behring, Deerfield, Illinois, USA), and 0.4 mM Z-Gly-Gly-Arg-AMC (Bachem, Torrance, California, USA) in HBS. After mixing, 100 µL was transferred to a FluoroNunc microtiter plate at 37°C to monitor fluorescence (excitation at 360 nm/emission at 460 nm; Gemini EM fluorescent plate reader (Molecular Devices, Sunnyvale, California, USA)). Fluorescence time course data were converted to nM thrombin as described [15]. ETP, defined as the area under the curve, was determined using Prism 5.04 (Graphpad, Software, San Diego, California, USA).

### FVa inactivation assays

APC-mediated inactivation of FVa was analyzed in ETP assays and aPTT clotting assays. FVa mutants were incubated with equal volumes of either rhAPC or buffer in human or murine BALB/c plasma.

### APTT clotting assays

Plasma (50 µL) was mixed with 50 µL of aPTT reagent (APTT-XL, Pacific Hemostasis, Thermo Fisher Scientific Inc., Waltham, Massachusetts, USA) and incubated at 37°C for 3 minutes in the presence of FVa and rhAPC. The clotting time was recorded using an ST4 coagulometer (Diagnostica Stago, Parsippany, New Jersey, USA) following the addition of 25 µL CaCl_2_ (50 mM) in HBS 0.5% BSA.

### Animals

All described animal protocols were carried out as approved by the institutional animal and care committee of The Scripps Research Institute. Female BALB/c mice, aged ≥8 weeks were used for experimentation. In case of factor VIII-deficient hemophilia A mice (BALB/c background; generous gift of Dr. David Lillicrap), mice of both genders aged ≥8 weeks were used.

### Ex-vivo aPTT clotting assays

BALB/c mice were administered rmAPC intravenously by tail vein injection 2 minutes prior to retroorbital blood harvest in siliconized microcapillaries (75 µL) prefilled with 20 µL sodium-citrate (3.8%). Whole blood aPTT was performed immediately by mixing 50 µL of blood with 50 µL of aPTT reagent (APTT-XL) in the presence of FVa or buffer. Clotting time were recorded using an ST4 coagulometer after incubation at 37°C for 3 minutes and following addition of 25 µL CaCl_2_ (130 mM) in HBS 0.5% BSA.

### Tail clip bleeding assay

Mice were anesthetized with isoflurane 3%, placed on temperature controlled heating pads (37°C), and the distal portion of the tail was cut at 1.5 mm diameter after which the tail was immersed in a predefined volume of 37°C saline (0.9% NaCl) for 20 minutes. To study effects on bleeding and clot stability, tubes were changed after 10 minutes to collect blood for the first and second 10 minutes separately. Blood loss was determined by the hemoglobin concentration in the saline solution after red cell lysis with 2% acetic acid and measured by absorbance at 490 nm. Using a hemoglobin standard derived from defined blood volumes, blood loss was calculated assuming a hematocrit of 46% and expressed in µL/g body weight. Groups of BALB/c mice were injected intravenously (retroorbital) with ^super^FVa or saline (200 µL) 2 minutes prior to intravenous (retroorbital) injection of rhAPC. Immediately after APC injection tail cut was performed. All agents were diluted in sterile sodium chloride 0.9% for injection (Hospira Inc, San Diego, California, USA). In some experiments rhFVIII (Xyntha, Pfizer) was injected intravenously at 200 U/kg.

### Liver laceration bleeding assay

Mice were anesthetized with Isofluorane 3% and the abdomen was opened by substernal blunt midline dissection. The liver was mobilized and externalized onto sterile gauze, followed by a defined 10 mm scalpel cut through the left liver lobe, which resulted in complete ventral and dorsal laceration. Immediately after laceration, mice were positioned prone into a small weighing dish (8 cm diameter) filled with saline (37°C, 13 mL) and transferred into the anesthesia chamber which rested on a heating pad (37°C). Anesthesia was maintained at 3% Isofluorane and dishes were changed after 10 minutes to collect blood for the first and second 10 minutes separately. Blood loss was determined as described for the tail clip model. Groups of BALB/c mice were injected intravenously (tail vein) with equal volumes (200 µL) of ^super^FVa or saline 2 minutes prior to intravenous injection of plasma-derived human APC, followed immediately by liver laceration. All agents were diluted in sterile sodium chloride 0.9% for injection (Hospira Inc). In some experiments rhFVIII (Xyntha, Pfizer) was injected intravenously at 200 U/kg.

### Statistical analysis

ANOVA with Bonferroni's multiple comparison test or for bleeding, Kruskal–Wallis followed by one-tailed Mann–Whitney test was used to assess statistical significance where appropriate. A P-value of ≤0.05 was considered statistically significant.

## Results

### Normalization of coagulation by ^super^FVa in the presence of APC

**Figure 1:**
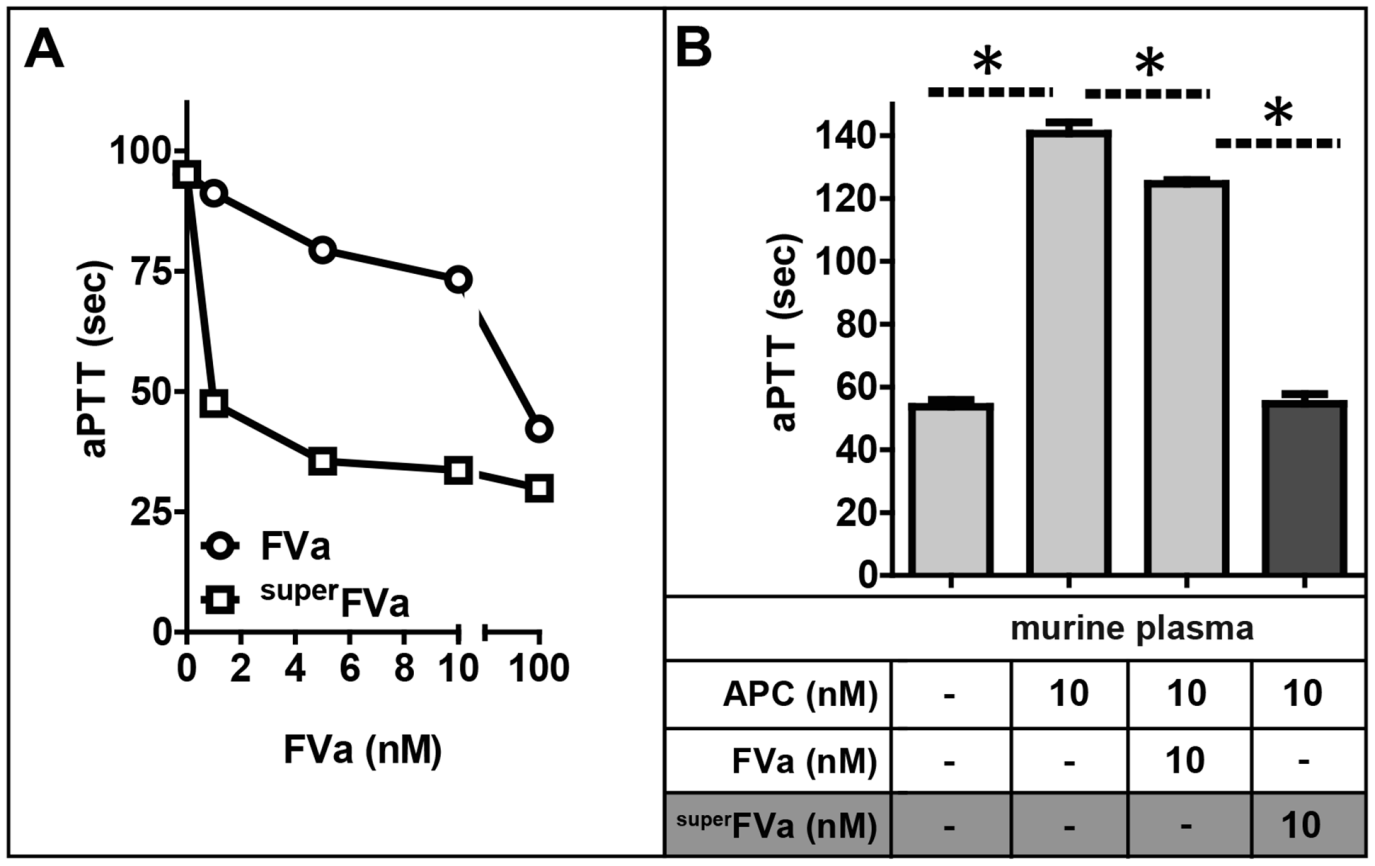
Correction of APC-inhibited coagulation by ^super^FVa and wt-FVa. **A**) APTT clotting times were determined in normal human plasma in the presence of 10 nM rhAPC and increasing concentrations of wt-FVa or ^super^FVa (n = 3). **B**) Comparison of normalization of APC inhibited coagulation by wt-FVa and ^super^FVa in murine plasma (n = 3). Error bars represent standard error of the mean. * denotes statistical significance (all p-values <0.001).

The ability of ^super^FVa to normalize APC-inhibited coagulation was initially determined by activated partial thromboplastin time (aPTT) clotting times. Dose-response titrations of APC in human and mouse plasma indicated the concentration of APC required for a prolongation of the aPTT to ∼100 seconds to be 10 nM **(Figure S1)**. Both ^super^FVa and wt-FVa dose-dependently normalized the aPTT in normal human plasma in the presence of 10 nM APC **(** **Figure 1a** **)**. There was an approximately two orders of magnitude difference in efficacy between ^super^FVa and wt-FVa, which cannot be explained by the approximately 1.5 to 3-fold higher specific activity of ^super^FVa in the prothrombinase assay [1]. This result suggests that the APC cleavage site mutations in ^super^FVa were likely responsible for the increased efficacy of ^super^FVa to normalize aPTT clotting times. Similar findings were evident in murine plasma. At a concentration of 10 nM, ^super^FVa corrected APC-induced aPTT prolongation, whereas wt-FVa achieved only partial shortening of the aPTT from ∼140 to 125 seconds **(** **Figure 1b** **)**.

**Figure 2:**
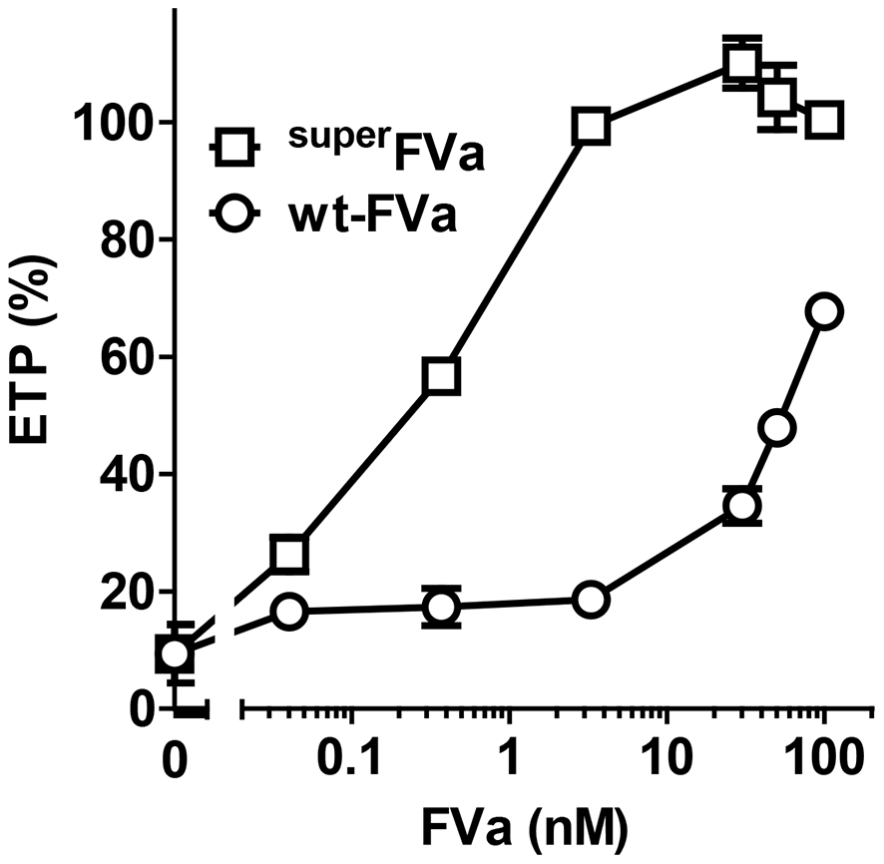
Correction of APC-inhibited thrombin generation by ^super^FVa and wt-FVa. Thrombin generation was determined in normal human plasma at increasing concentrations of ^super^FVa or wt-FVa in the presence of rhAPC (5 nM). Thrombin generation was expressed as the relative change in the endogenous thrombin potential (ETP) in the absence of APC. Error bars represent standard error of the mean (n = 3–4).

The ability of ^super^FVa to normalize coagulation in the presence of APC was also characterized by analyzing thrombin generation in human plasma as determined by the endogenous thrombin potential (ETP). Suppression of ETP in human plasma by APC was found to be optimal at APC concentrations of 5 nM **(Figure S2)**. When thrombin generation was suppressed by APC (5 nM), ^super^FVa at 2.5 nM restored ETP to ∼100% of normal, whereas in the presence of wt-FVa at 100 nM the ETP was restored to only ∼70% of normal **(** **Figure 2** **)**. To compare and contrast the effects of ^super^FVa to other prohemostatic agents used to arrest bleeding, the ability of recombinant human (rh)FVIIa and 4-Factor Prothrombin Complex Concentrate (PCC) to normalize APC-inhibited thrombin generation were determined. Dose-response titrations of rhFVIIa up to 2 µg/ml, which corresponds to an extrapolated therapeutic dose of 90 µg/kg (based on a general plasma volume of ∼45 ml/kg) did not increase thrombin generation in the presence of 5 nM APC **(Figure S3)**. Similarly, dose-response titrations of PCC up to 1.5 U/ml (corresponding to extrapolated therapeutic dose of 25–50 U/kg) were unable to correct thrombin generation in the presence of APC **(Figure S4)**. In comparison, dose-response titrations of ^super^FVa indicated normalization of ETP at >3 nM ^super^FVa and partial normalization of thrombin peak height **(Figure S5)**. Thus, currently available prohemostatic agents used clinically in all cause catastrophic bleeding situations are relatively ineffective to correct APC-mediated inhibition of coagulation.

### Correction of ex vivo clotting times by ^super^FVa after in vivo administration of APC in mice

**Figure 3:**
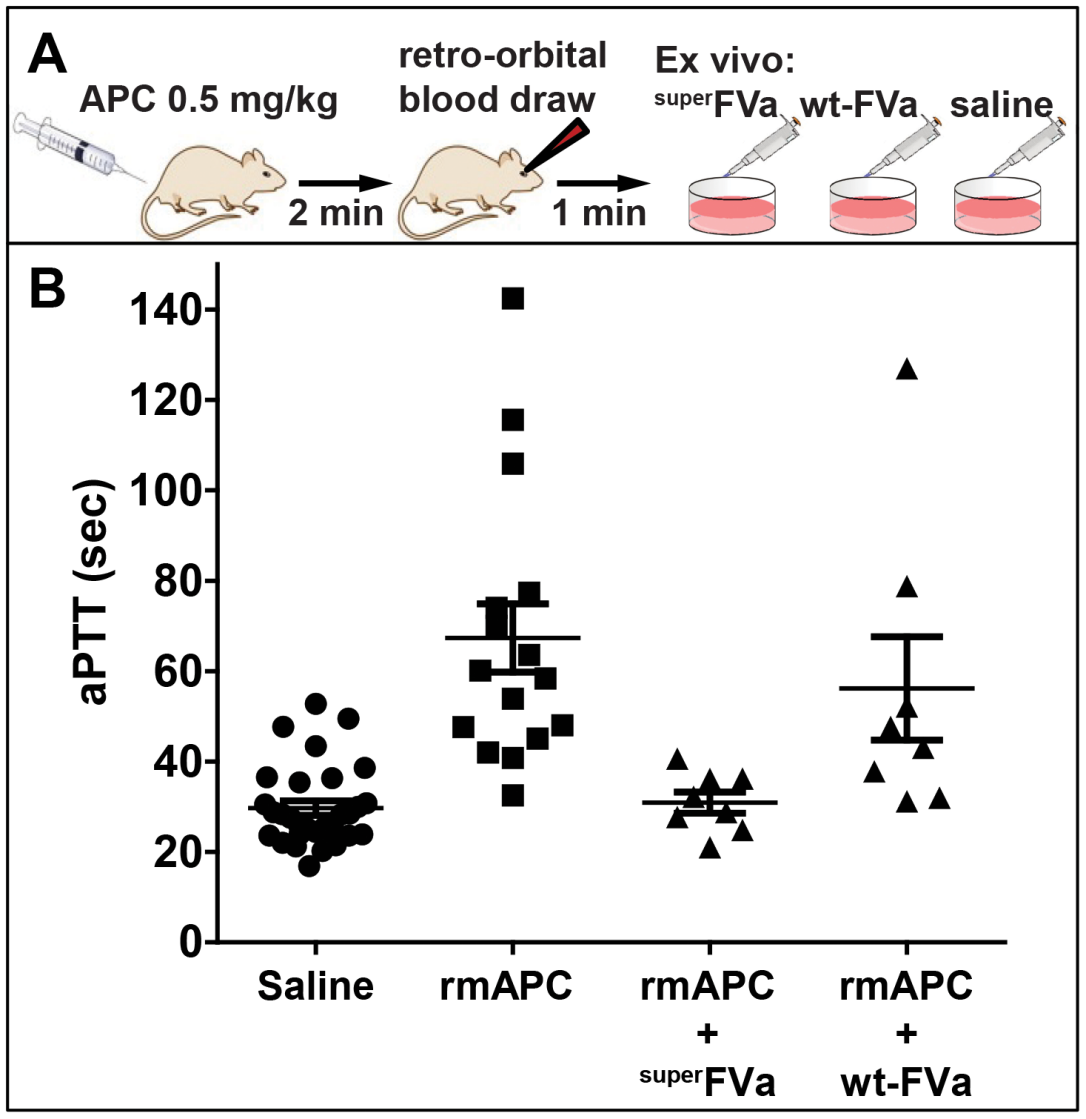
Correction of aPTT by FVa variants ex vivo after in vivo APC administration. **A**) Experimental schematic. BalbC mice were injected intravenously with saline or recombinant murine APC (0.5 mg/kg). Blood was collected by retro-orbital blood draw two minutes later. **B**) Blood of mice injected with APC was spiked ex vivo with either saline, ^super^FVa or wt-FVa (both 1 nM) and whole blood aPTT was determined immediately. Error bars represent standard error of the mean.

Initially, a combined in vivo/ex vivo approach was chosen to determine prolongation of aPTT clotting times in mouse blood by APC, and to probe the ability of wt-FVa or ^super^FVa to shorten APC-prolonged clotting times. Recombinant murine (rm)APC was administered in vivo and inhibition of coagulation was determined by whole blood aPTT assays after ex vivo addition of wt-FVa or ^super^FVa. Due to the short in vivo half-life of APC (∼16 minutes [16]), whole blood aPTT performed within minutes after blood harvest was used as opposed to plasma that takes longer to prepare. APTT clotting times doubled (30 sec saline (n = 31); 67 sec rmAPC (n = 16); p<0.05) in blood immediately drawn after intravenous injection of rmAPC (0.5 mg/kg) in BALB/c mice **(** **Figure 3** **)**. Ex vivo addition of ^super^FVa to whole blood (31 sec ^super^FVa (n = 8); p<0.05) normalized the aPTT to that in the absence of rmAPC. In contrast, ex vivo addition of a similar activity-based dose of wt-FVa provided only a partial normalization of the aPTT (56 sec wt-FVa (n = 8)).

### Prevention of acute APC-induced bleeding by ^super^FVa

Two different in vivo models, tail clip and liver laceration, were employed to study the extent to which ^super^FVa could reduce bleeding following large vessel as well as parenchymal microvessel injury.

**Figure 4:**
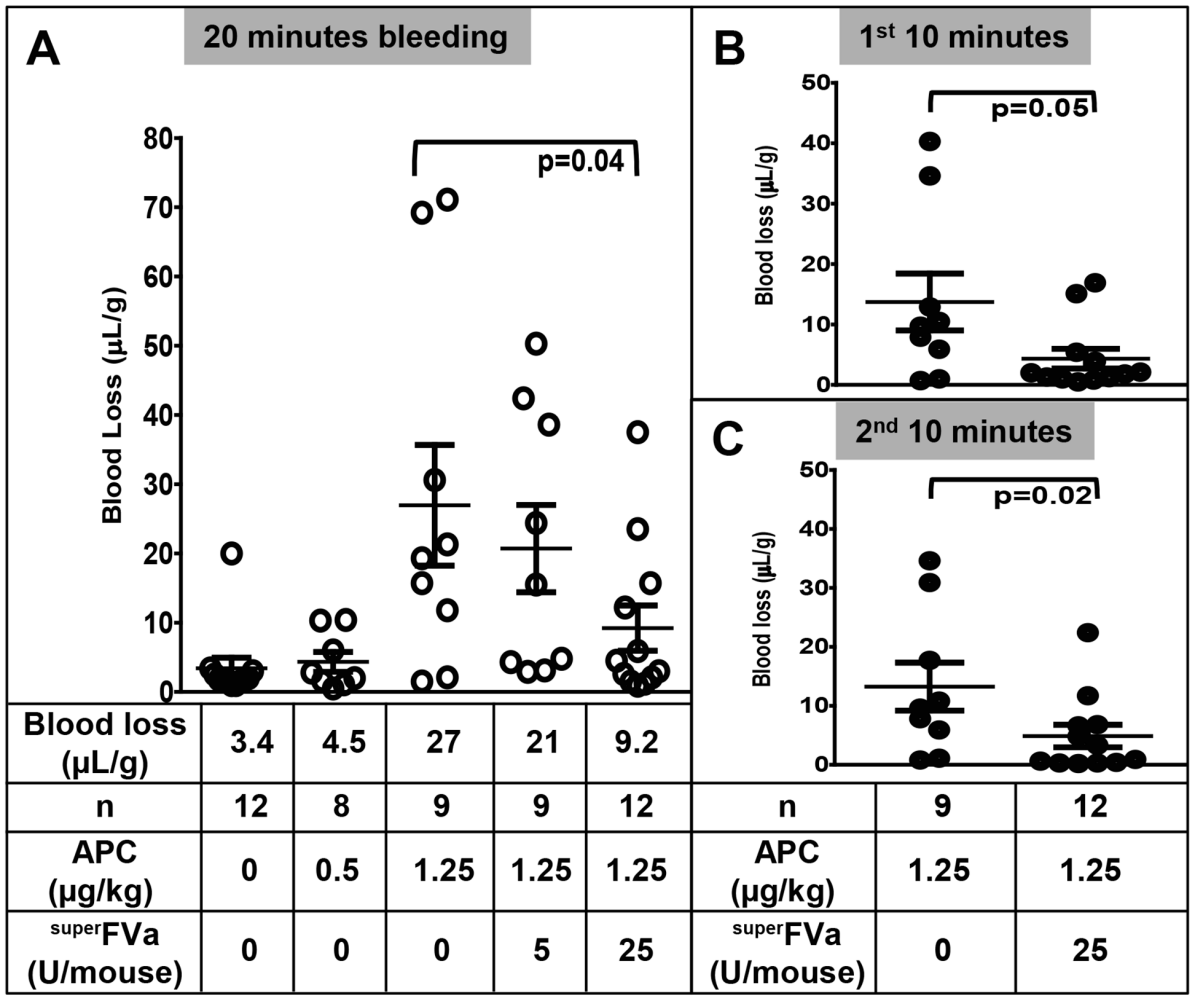
Correction of APC-induced bleeding by superFVa in the murine tail clip model. Wild-type BalbC mice were injected intravenously with increasing doses of rhAPC or with saline. ^Super^FVa was injected intravenously 2 minutes prior to APC. Bleeding after tail clip is expressed as blood loss in µl blood per gram mouse. **A**) Blood loss during 20 minutes, and divided into **B**) first 10 minutes and **C**) second 10 minutes after tail clip. Error bars represent SEM.

The tail clip model was used to characterize the reversal of acute APC-induced bleeding following large vessel transection by ^super^FVa in vivo. To determine the dose of APC required to induce notable bleeding rhAPC was administered at 0.5 mg/kg and 1.25 mg/kg **(** **Figure 4a** **)**. Notable bleeding was achieved only at the higher concentration of rhAPC (1.25 mg/kg), which corresponds to our vitro findings demonstrating that the concentration of human APC required to reduce thrombin generation in murine plasma is at least 20-fold higher than in human plasma **(Figure S2)**. This concentration is also within the expected range of rhAPC effects in murine plasma and mouse studies [7], [17], where it has 6-fold less anticoagulant effects than in human plasma [18]. Blood was collected after tail clip for the first 10 min and second 10–20 min separately to distinguish initial bleeding from late rebleeding. At 1.25 mg/kg, APC blood loss increased from 3.4 µL/g (saline) to 27 µL/g in APC treated mice during the combined 20 minute bleeding period **(** **Figure 4a** **)**. Administration of ^super^FVa two minutes prior to APC-injection decreased blood loss dose-dependently. At 25 U/mouse ^super^FVa APC-induced blood loss was reduced to 9.2 µL/g (p = 0.04) **(** **Figure 4a** **)**. Blood loss following APC was continuous and similar during the first and second 10 minutes after tail clip (mean 13.7 and 13.2 µL/g, respectively). ^Super^FVa (25 U/mouse) reduced blood loss during both periods, from 13.7 to 4.3 µL/g during the first 10 min **(** **Figure 4b** **)** and from 13.2 to 4.9 µL/g during the second 10 minutes **(** **Figure 4c** **)**.

### Validation of a liver laceration model in mice to study microvascular parenchymal organ bleeding

To provide additional support for reversal of APC-induced bleeding by ^super^FVa, a liver laceration model was introduced to mouse studies and validated in hemophilia A mice by side-by-side comparison to the tail transection model. Liver laceration is frequently used as a bleeding model in larger animals such as rats, rabbits and swine since it provides important information on microvascular-mediated parenchymal profuse bleeding after acute traumatic organ injury [19]–[21]. In contrast, bleeding after tail clip emulates bleeding patterns following complete transection of larger arterial and venous vessels. Bleeding patterns in both models may differ and may provide complementary information. However, liver laceration is rarely performed in mice and not universally established due to technical challenges pertaining to surgical intervention and abdominal blood collection in small animals. Here, a surgical approach was established modified from Bajaj et al. [22], whereby the liver is externalized after abdominal midline incision, and the left liver lobe is lacerated with a 10 mm long scalpel cut, followed by blood collection with the mouse in prone position into 37°C saline (see **Figure S6** for a photographic documentation of the methodology).

**Figure 5:**
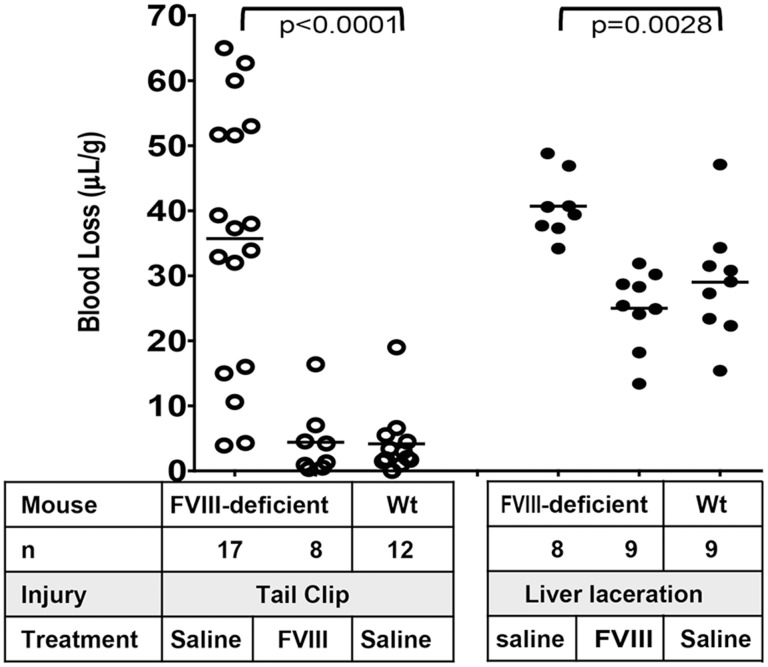
Comparison of tail clip and liver laceration bleeding models. FVIII-deficient mice were injected intravenously with saline or rhFVIII (50 U/kg) and subjected to tail clip or liver laceration. Wt-mice were injected with saline. Blood loss was determined during 20 minutes and expressed in µL per gram mouse. Horizontal lines represent mean blood loss.

To provide validation of the liver laceration model as a method to quantify bleeding, its results were compared to that obtained in the tail clip model in hemophilia A mice, an established bleeding diathesis model for hemophilia. Blood loss after 20 minutes was similar in both models (tail clip 35.7 µL/g; liver laceration 40.7 µL/g). Unlike with tail clip, where some hemophilia mice do not exhibit prominent bleeding, thereby resulting in wide inter-individual bleeding ranges, liver laceration invariably caused bleeding in all mice **(** **Figure 5** **)**. Treatment of hemophilia A mice with rhFVIII (200 U/kg) reduced bleeding in both models to that observed in wt-BALB/c mice treated with saline control. In the liver laceration FVIII reduced bleeding in hemophilia mice from 40.7 µL/g to 25.0 µL/g (versus 29.0 µL/g in saline-treated wt-BALB/c mice) and in the tail clip model FVIII reduced bleeding from 35.7 µL/g to 4.4 µL/g (versus 4.2 µL/g in saline-treated wt-BALB/c mice). These results validate the liver laceration model as an additional method to study hemostasis in mice.

### Bleed prevention and mortality rescue by ^super^FVa upon APC-induced bleeding after liver laceration

**Figure 6:**
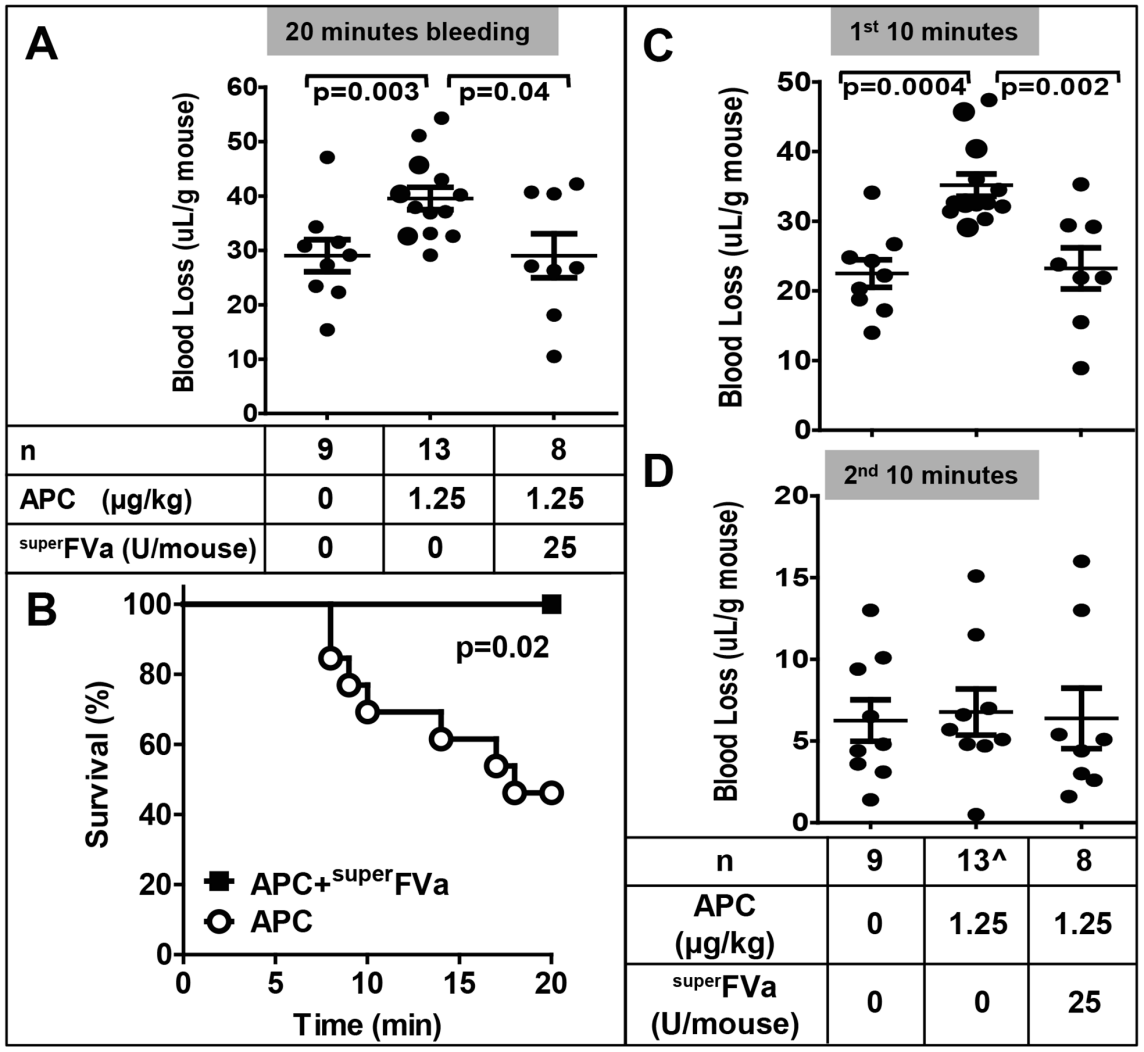
Correction of APC-induced fatal bleeding by superFVa after liver laceration. Mice were injected intravenously with saline or plasma derived human APC at 1.25 mg/kg. ^Super^FVa was injected intravenously 2 minutes prior to APC. Bleeding after liver laceration is expressed as blood loss in µl blood per gram mouse. **A**) Blood loss during 20 minutes and **B**) survival. **C**) Blood loss divided into first 10 minutes and **D**) second 10 minutes after injury. **∧** denotes that four of 13 mice injected with APC died during the first 10 minutes after injury and are therefore excluded from the bottom panel. Error bars represent standard error of the mean.

To induce APC-mediated bleeding in the liver laceration model, wt-BALB/c were injected intravenously with recombinant human wt-APC (1.25 mg/kg) based on the increased bleeding that this dose of APC caused in the tail bleed model. Wt-APC administration increased bleeding after liver laceration (mean blood loss 39.5 µL/g APC compared to 29.0 µL/g saline; p = 0.003) over 20 min **(** **Figure 6a** **)**. Treatment of APC-induced bleeding with ^super^FVa (29.0 µL/g; p = 0.04) decreased blood loss to baseline values similar to that observed in non-APC treated mice **(** **Figure 6a** **)**. APC-treatment in the liver laceration model caused excessive bleeding during the first 10 minutes which was associated with a ∼50% mortality rate. Four mice died during the first 10 min and an additional 3 mice died during the second 10 min **(** **Figure 6b** **)**. Treatment with ^super^FVa (25 U/mouse) provided full mortality rescue. Blood loss during the first 10 minutes after liver laceration **(** **Figure 6c** **)** was pronounced following APC-injection (35.1 µL/g vs. saline 22.5 µL/g; p = 0.0004), whereas blood loss during the second 10 min was minimal and not increased by APC **(** **Figure 6d** **)**. These results highlight that parenchymal bleeding is different from tail bleeding and can provide additional information as to prevention of fatal exsanguination with certain treatments.

## Discussion

Severe hemorrhage is a frequent complication of anticoagulant therapy in general [23], has been reported with APC-therapy [6] and may complicate clinical development of wt-APC for important indications such as mitigation of radiation injury [24]. Another area where APC contributes to bleeding is acute traumatic coagulopathy, driven by the combination of tissue injury and shock and characterized by global endogenous activation of the protein C and fibrinolytic pathways. This condition is often associated with uncontrollable hemorrhage, increased mortality, and worse outcome in the polytrauma patient [25]–[27].

Here, we demonstrate that ^super^FVa, an engineered FVa variant, is an efficient prohemostatic reversal agent for bleeding induced by wt-APC. The biochemical characterization of ^super^FVa and its efficacy of bleed control in a hemophilic mouse model after tail clip was previously published [1]. Because B-domain deleted FV has some inherent cofactor activity, only activated FVa's were compared. Furthermore, it was previously reported that clot formation with human plasma derived FV in hemophilia mice required prior activation of the FV [28]. In vitro, ^super^FVa normalized APC-induced prolongation of the aPTT at ∼100-fold lower concentration than FVa in human plasma, and resulted in correction of ETP at concentrations where FVa, or currently available prohemostatic reversal agents (rhFVIIa and PCC) did not show any effects. Similar results were obtained for ^super^FVa in murine plasma indicating that the mouse can be used to study the effects of ^super^FVa on bleeding diathesis in response to human APC.

Two different bleeding models, the conventional tail clip model and a newly introduced liver laceration model, were used to demonstrate ^super^FVa's efficacy as a reversal agent against APC-induced fatal bleeding. Usually liver laceration is performed in larger animals such as the rat, rabbit or and swine due to ease of anatomic access [19]–[21]. We were successful to adapt the procedure for the anatomy of the mouse [22], which provided the unique opportunity to study rescue from fatal bleeding. Comparison of the new liver laceration model with the established tail lip model in hemophilia A mice, with and without treatment with rhFVIII, provided validation and confidence for quantitative analysis of bleeding using liver laceration. While the tail clip model assesses the bleeding pattern following direct transection of large caliber arterial and venous vessels, liver laceration addresses parenchymal bleeding after traumatic organ injury that in humans can be catastrophic and lethal when occurring while on anticoagulant treatment [23]. Liver laceration as a model may also mimic more closely microvascular bleeding patterns as encountered during intramuscular or intracranial hemorrhage.

^Super^FVa reduced blood loss in APC-treated mice in both bleeding models providing in vivo proof-of-principle for ^super^FVa as a reversal agent for APC-induced bleeding. Interestingly, bleeding patterns were different in both models when blood loss was determined separately for the first and second 10 min after injury. APC-induced bleeding after tail clip was continuous over 20 min, and ^super^FVa decreased blood loss during both phases. Blood loss in the liver laceration model was massive and occurred immediately after injury with little bleeding during the second 10 min. This acute blood loss was associated with a ∼50% mortality rate. Notably, ^super^FVa not only provided significant bleed protection similar to blood loss in mice without APC treatment, but also abolished bleeding-induced mortality.

The mechanism by which ^super^FVa prevents APC-induced bleeding is consistent with FVa activity augmentation within the prothrombinase complex [1]. It is well described that the presence of FVa in the prothrombinase complex potently enhances the rate of thrombin generation to approximately 10,000-fold [2], [29]. However, FVa is also rapidly inactivated by APC via proteolytic cleavage at Arg506 followed by a slower cleavage at Arg306 [9]. Mutations of these inactivation cleavage sites, such as Arg506Gln (a.k.a. FV_Leiden_), extend the FVa cofactor activity half-life. In vivo relevance that FVa and especially mutations that render FVa resistant to inactivation by APC can mitigate clinical bleeding is derived from studies in hemophilic patients and mice. The FV_Leiden_ mutation is now widely accepted to be a disease modifier in persons with hemophilia and hemophilic mice [28], [30]. Moreover, the pharmacological administration of FVa to hemophilic mice was demonstrated to improve coagulation profiles as well as bleeding, and blood loss was much more efficiently abrogated by ^super^FVa [1], [28].

Continuous infusion (24 µg/kg/hr) of wt-APC for 96 h in severe sepsis patients was associated with an increased risk of bleeding in large phase III clinical trials [6], and wt-APC is currently no longer available for clinical use. Bleeding risks for second-generation APC-therapy currently in clinical development for ischemic stroke [31] are mitigated by mutations of the APC exosite that diminish APC's anticoagulant activity, and repeated bolus dosing rather than continuous infusion. However, APC plasma concentrations for this indication will exceed the steady-state levels of wt-APC in the sepsis trials, and may be as much as approximately 100-fold higher [31]. Additional indications for APC-treatment such as acute radiation injury may continue to require APC's anticoagulant function. Mortality reduction after lethal total body irradiation was demonstrated for wt-APC and anticoagulant-selective E149A-APC, but not for cytoprotective-selective 5A-APC [24]. Proof for the potential availability of an effective prevention and/or reversal agent for APC-induced bleeding, such as ^super^FVa, may therefore facilitate considerations for the development of APC-based therapies.

The experiments described here provide proof-of-principle that ^super^FVa is effective in the prevention and reversal of bleeding induced by wt-APC, thereby adding to the evidence that it has characteristics of a potent hemostatic agent. Strategies to prevent or stop severe bleeding are desirable in numerous other clinical situations where currently available hemostatic treatments are ineffective or suboptimal. For instance, it may be meritorious to study ^super^FVa in the setting of acute traumatic coagulopathy where recent evidence suggests that early bleeding may be exacerbated by endogenous APC generation [25]–[27], in hemophilia where inhibitor formation against exogenous FVIII or FIX requires alternative hemostatic treatments, or for reduction of bleeding associated with (novel oral) anticoagulants. In all those situations there is a paucity of therapeutic options available, of which most are of questionable efficacy. Reversal agents for these indications present an urgent and unmet clinical need. Whether ^super^FVa will be effective in all of these situations, much like a universal prohemostatic agent, will remain subject of future studies.

## Supporting Information

Figure S1 **Inhibition of aPTT clotting by APC.** Dose dependent prolongation of aPTT clotting times by rhAPC was determined in (A) normal human plasma and (B) murine plasma. APC; Activated Protein C. Error bars represent SEM.
(TIF)

Figure S2 **Inhibition of coagulation by APC.** Dose dependent suppression of thrombin generation by rhAPC was determined in (**A**) normal human plasma and (**B**) murine plasma. Thrombin generation was expressed as area under curve (AUC). APC; Activated Protein C.
(TIF)

Figure S3 **Reversal of APC-mediated inhibition of coagulation by rhFVIIa.** Thrombin generation was determined by the endogenous thrombin potential (ETP) and peak height (ETP_max_) in normal human plasma supplemented with rhFVIIa in the presence of 5 nM rhAPC. (**A**) Representative example of ETP with increasing concentrations of rhFVIIa. (**B**) ETP (top panel) and peak height (bottom panel) achieved with increasing concentrations of rhFVIIa. NHP; normal human plasma. Error bars represent standard error of the mean (n≥3).
(TIF)

Figure S4 **Reversal of APC-mediated inhibition of coagulation by 4-Factor Prothrombin Complex Concentrate.** Thrombin generation was determined by the endogenous thrombin potential (ETP) and peak height (ETP_max_) in normal human plasma supplemented with 4-Factor Prothrombin Complex Concentrate (PCC) in the presence of 5 nM rhAPC. (**A**) Representative example of ETP with increasing concentrations of PCC. (**B**) ETP (top panel) and peak height (bottom panel) achieved with increasing concentrations of PCC. NHP; normal human plasma. Error bars represent standard error of the mean (n≥3).
(TIF)

Figure S5 **Reversal of APC-mediated inhibition of coagulation by ^super^FVa.** Thrombin generation was determined by the endogenous thrombin potential (ETP) and peak height (ETP_max_) in normal human plasma supplemented with ^super^FVa in the presence of 5 nM rhAPC. (**A**) Representative example of ETP with increasing concentrations of ^super^FVa. (**B**) ETP (top panel) and peak height (bottom panel) achieved with increasing concentrations of ^super^FVa. APC; Activated protein C. NHP; normal human plasma. Error bars represent standard error of the mean (n≥3).
(TIF)

Figure S6 **Photographic illustration of the liver laceration model.** Mice were anesthesized (**A**) and the upper abdomen was opened via substernal midline dissection (**B**). The liver was carefully externalized with blunt tweezers (**C**) and the left liver lobe was transected with a scalpel (**D,E**). The scalpel was marked to create a precise 10 mm cut. During the photography the gauze became blood soaked, which is not the case during the real procedure where the mouse is positioned prone into saline (13 mL) seconds after liver laceration, followed by transfer into the anesthesia chamber (**F,G**). All procedures were carried out under a heating lamp. The saline temperature was 37°C. A heating pad was positioned underneath the anesthesia chamber for the duration of the 20 min observation period (not shown). Hemoglobin quantification in the saline was performed as described in Material and Methods.
(TIF)
