# Supplementary figures and images for: An Engineered Factor Va Prevents Bleeding Induced by Anticoagulant wt Activated Protein C
Source: PLoS One. 2014 Aug 15;9(8):e104304. doi: 10.1371/journal.pone.0104304 (PMC4134195; doi:10.1371/journal.pone.0104304)

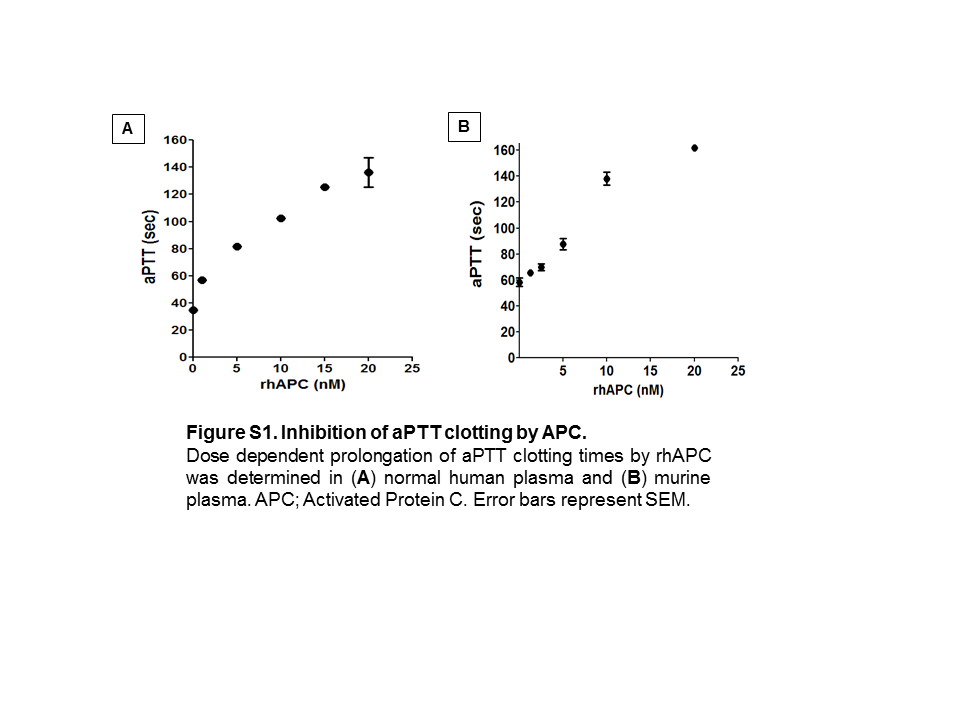

Supplement: Figure S1 — Inhibition of aPTT clotting by APC. Dose dependent prolongation of aPTT clotting times by rhAPC was determined in (A) normal human plasma and (B) murine plasma. APC; Activated Protein C. Error bars represent SEM. (TIF) [file pone.0104304.s001.tif]

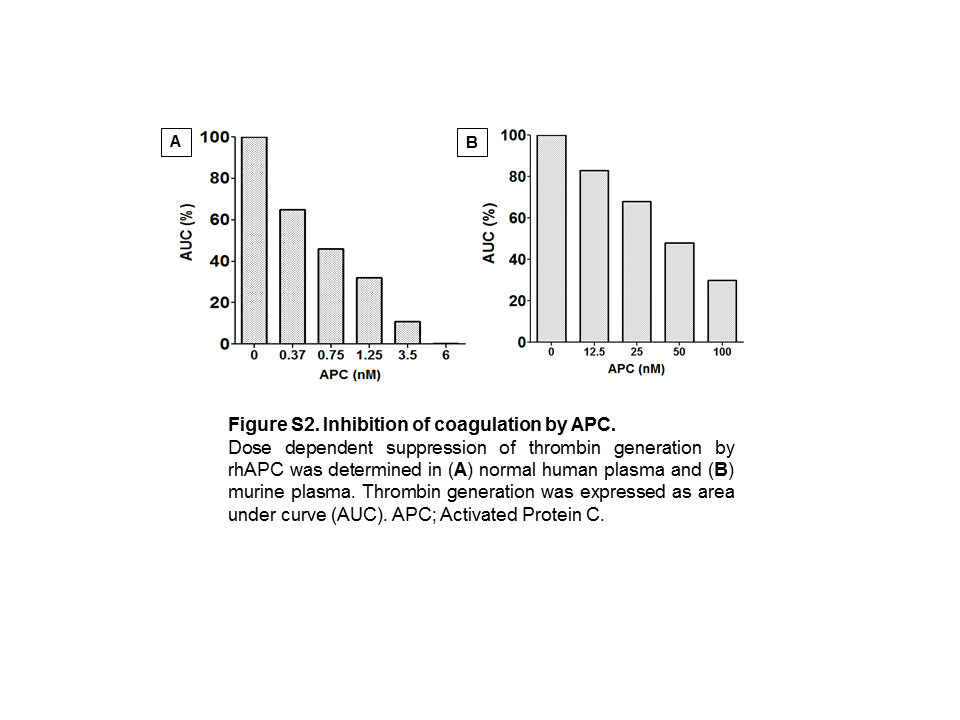

Supplement: Figure S2 — Inhibition of coagulation by APC. Dose dependent suppression of thrombin generation by rhAPC was determined in (A) normal human plasma and (B) murine plasma. Thrombin generation was expressed as area under curve (AUC). APC; Activated Protein C. (TIF) [file pone.0104304.s002.tif]

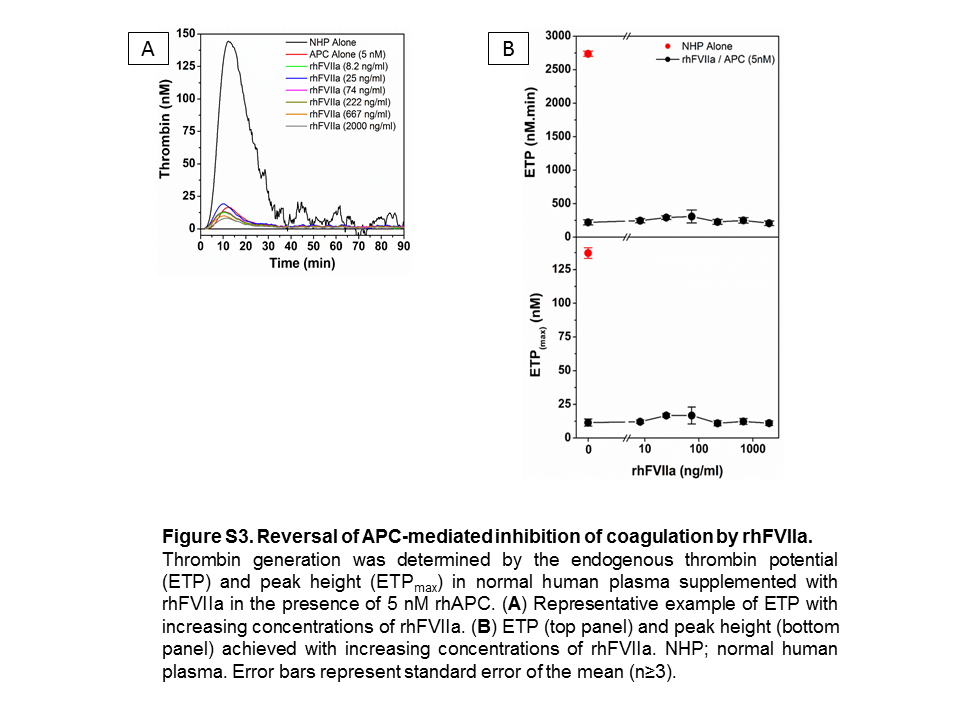

Supplement: Figure S3 — Reversal of APC-mediated inhibition of coagulation by rhFVIIa. Thrombin generation was determined by the endogenous thrombin potential (ETP) and peak height (ETPmax) in normal human plasma supplemented with rhFVIIa in the presence of 5 nM rhAPC. (A) Representative example of ETP with increasing concentrations of rhFVIIa. (B) ETP (top panel) and peak height (bottom panel) achieved with increasing concentrations of rhFVIIa. NHP; normal human plasma. Error bars represent standard error of the mean (n≥3). (TIF) [file pone.0104304.s003.tif]

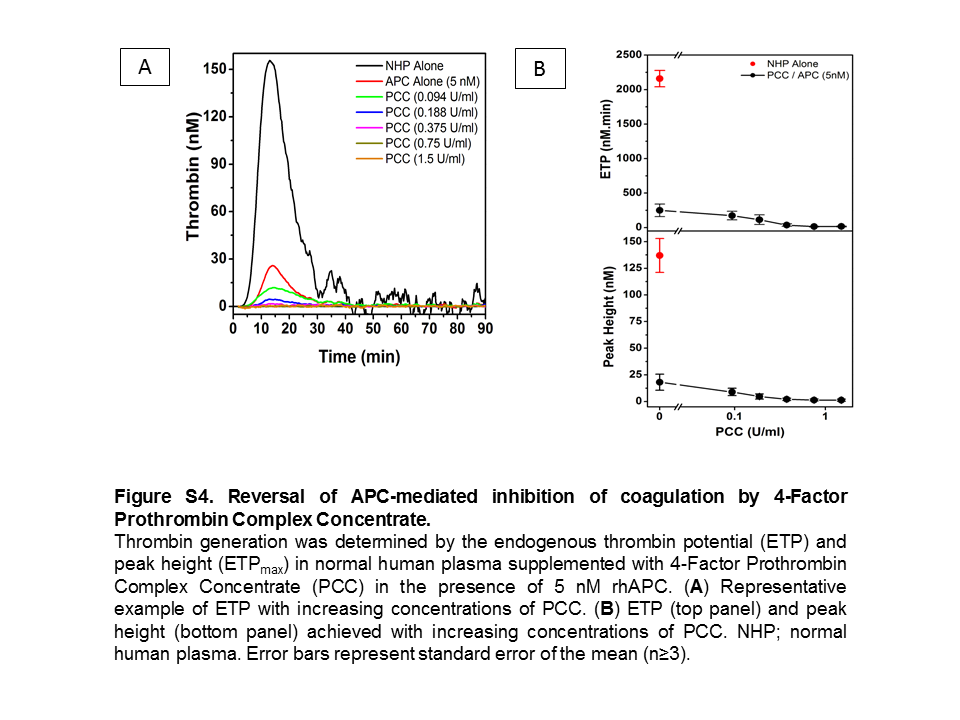

Supplement: Figure S4 — Reversal of APC-mediated inhibition of coagulation by 4-Factor Prothrombin Complex Concentrate. Thrombin generation was determined by the endogenous thrombin potential (ETP) and peak height (ETPmax) in normal human plasma supplemented with 4-Factor Prothrombin Complex Concentrate (PCC) in the presence of 5 nM rhAPC. (A) Representative example of ETP with increasing concentrations of PCC. (B) ETP (top panel) and peak height (bottom panel) achieved with increasing concentrations of PCC. NHP; normal human plasma. Error bars represent standard error of the mean (n≥3). (TIF) [file pone.0104304.s004.tif]

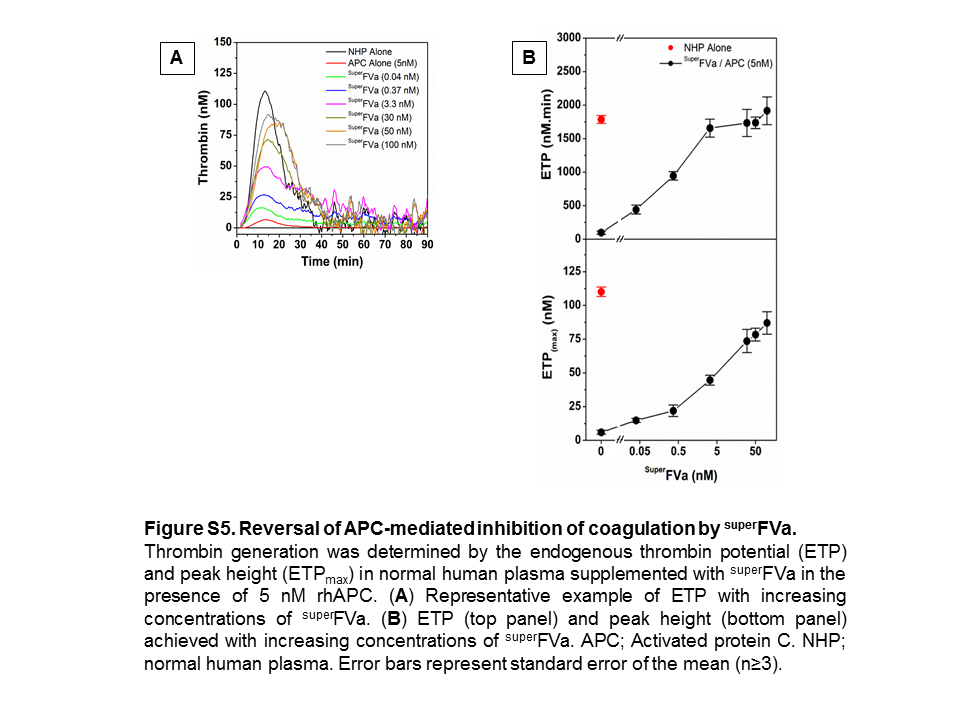

Supplement: Figure S5 — Reversal of APC-mediated inhibition of coagulation by superFVa. Thrombin generation was determined by the endogenous thrombin potential (ETP) and peak height (ETPmax) in normal human plasma supplemented with superFVa in the presence of 5 nM rhAPC. (A) Representative example of ETP with increasing concentrations of superFVa. (B) ETP (top panel) and peak height (bottom panel) achieved with increasing concentrations of superFVa. APC; Activated protein C. NHP; normal human plasma. Error bars represent standard error of the mean (n≥3). (TIF) [file pone.0104304.s005.tif]

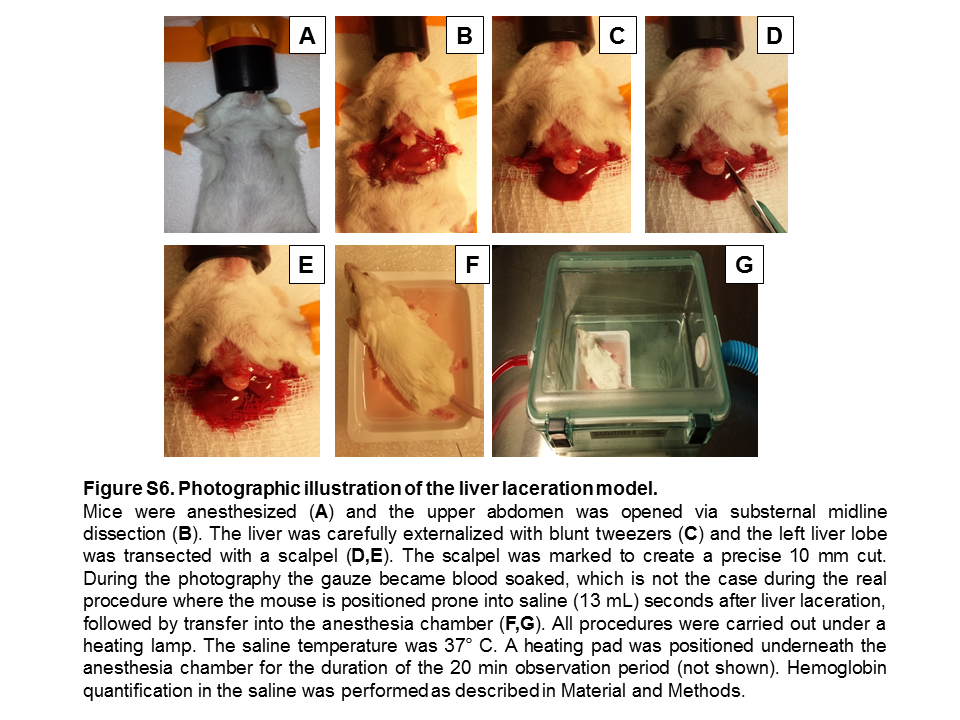

Supplement: Figure S6 — Photographic illustration of the liver laceration model. Mice were anesthesized (A) and the upper abdomen was opened via substernal midline dissection (B). The liver was carefully externalized with blunt tweezers (C) and the left liver lobe was transected with a scalpel (D,E). The scalpel was marked to create a precise 10 mm cut. During the photography the gauze became blood soaked, which is not the case during the real procedure where the mouse is positioned prone into saline (13 mL) seconds after liver laceration, followed by transfer into the anesthesia chamber (F,G). All procedures were carried out under a heating lamp. The saline temperature was 37°C. A heating pad was positioned underneath the anesthesia chamber for the duration of the 20 min observation period (not shown). Hemoglobin quantification in the saline was performed as described in Material and Methods. (TIF) [file pone.0104304.s006.tif]
